# How a Spatial Arrangement of Secondary Structure Elements Is Dispersed in the Universe of Protein Folds

**DOI:** 10.1371/journal.pone.0107959

**Published:** 2014-09-22

**Authors:** Shintaro Minami, Kengo Sawada, George Chikenji

**Affiliations:** 1 Department of Complex Systems Science, Nagoya University, Nagoya, Aichi, Japan; 2 Department of Applied Physics, Nagoya University, Nagoya, Aichi, Japan; 3 Department of Computational Science and Engineering, Nagoya University, Nagoya, Aichi, Japan; University of Michigan, United States of America

## Abstract

It has been known that topologically different proteins of the same class sometimes share the same spatial arrangement of secondary structure elements (SSEs). However, the frequency by which topologically different structures share the same spatial arrangement of SSEs is unclear. It is important to estimate this frequency because it provides both a deeper understanding of the geometry of protein folds and a valuable suggestion for predicting protein structures with novel folds. Here we clarified the frequency with which protein folds share the same SSE packing arrangement with other folds, the types of spatial arrangement of SSEs that are frequently observed across different folds, and the diversity of protein folds that share the same spatial arrangement of SSEs with a given fold, using a protein structure alignment program MICAN, which we have been developing. By performing comprehensive structural comparison of SCOP fold representatives, we found that approximately 80% of protein folds share the same spatial arrangement of SSEs with other folds. We also observed that many protein pairs that share the same spatial arrangement of SSEs belong to the different classes, often with an opposing N- to C-terminal direction of the polypeptide chain. The most frequently observed spatial arrangement of SSEs was the 2-layer *α*/*β* packing arrangement and it was dispersed among as many as 27% of SCOP fold representatives. These results suggest that the same spatial arrangements of SSEs are adopted by a wide variety of different folds and that the spatial arrangement of SSEs is highly robust against the N- to C-terminal direction of the polypeptide chain.

## Introduction

The protein fold is defined by the number, spatial arrangement, and topological connectivity of secondary structure elements (SSEs) [1]. Currently existing protein folds resulted from both physicochemical interactions and evolutionary selection. The recent accumulation of tens of thousands of protein structures and the development of various sensitive sequence/structure alignment algorithms have helped to provide significant insights into several aspects concerning protein folds, such as their diversity [1]–[3], the evolutionary mechanism of fold change [4]–[6], the discreteness and continuity of the fold space [7]–[9], and the shape of the protein fold universe [10], [11]. However, some aspects of the physics or geometry of protein folds, such as the nature of spatial arrangement of SSEs, remain less understood. In this paper, we studied the geometrical aspects of protein structures by focusing on the spatial arrangement of SSEs. As the primary subject of this study, we should clarify our definition of the term “spatial arrangement of SSEs.” This term will imply the relative atomic positions of the backbone atoms within SSEs for which their connectivity and the N- to C- terminal direction are ignored.

To gather insights into the nature of the spatial arrangement of SSEs in the organization of protein folds, we primarily addressed the following three questions. The first question is “How many protein folds share the same SSE packing arrangement with at least one other fold?” It is known that some protein pairs with clearly different folds share the same spatial arrangement of SSEs [4], [12]–[14]. In contrast, it was also reported that some other folds have a unique spatial arrangement of SSEs and do not display any structural similarity to other folds even if the chain connectivity is ignored [15]. However, the frequency by which different folds share the same spatial arrangement of SSEs is unclear. It is important to estimate this frequency because it provides both an insight into the completeness of the secondary structure packing pattern and an estimation of the upper limit of the prediction success of rewiring or multiple loop permutation for predicting protein structures with novel folds [16]–[18]. The second question is “What types of SSE spatial arrangements are frequently observed across different folds?” As previously described, some spatial arrangements of SSEs are observed only in one fold type [15]. For these folds, the particular connectivity of SSEs, which is closely related to the local interactions along the chain in the loop regions, may be essential for adopting such a particular fold. Conversely, it has been demonstrated that some spatial arrangements of SSEs are observed in many different folds with different SSE connectivities [16], [19]–[22]. For these folds, non-local interactions may play a dominant role in maintaining the fold structure. Thus, identifying what types of SSE spatial arrangements are rarely or frequently observed in the protein fold space would provide an insight into the relative importance of local versus non-local interactions in the organization of protein folds [23], [24]. The third question is “How diverse are the protein folds that share the same spatial arrangement of SSEs with a given fold?” It is well known that different folds of the same SCOP class often share the same spatial arrangement of SSEs [16], [25]. However, it remains unclear how often protein folds belonging to different SCOP classes share the same spatial arrangement of SSEs. It is interesting to examine this issue because it would provide a deeper understanding of the universality or generality of secondary structure packing patterns.

**Figure 1:**
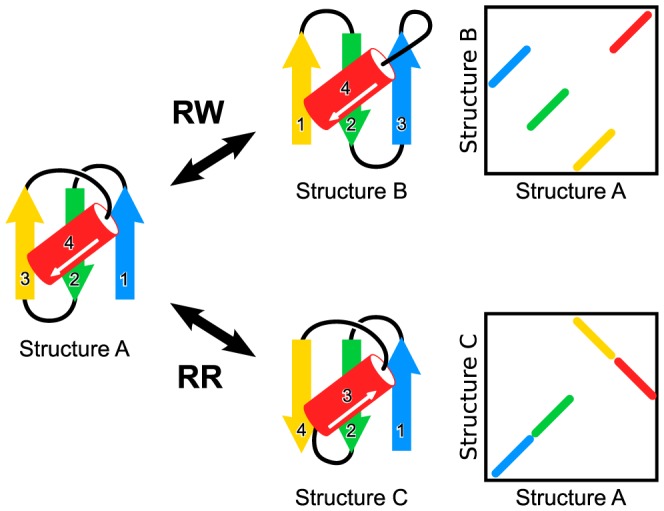
Schematic examples of structure alignment by the RW and RR alignment schemes. The protein structure is shown as a schematic model. Structures A and B were aligned by the RW scheme, and structures A and C were aligned by the RR scheme. The corresponding alignment plots are shown on the right hand side of figure.

To answer these questions, we must perform structure comparisons and identify the protein pairs that share the same spatial arrangement of SSEs. One of the algorithms that can detect the same spatial arrangement of SSEs is a non-sequential structure alignment algorithm in which structurally equivalent SSEs are aligned in different orders in the protein sequences (rewiring) and permitted to have the opposite N- to C-terminal direction of SSEs (reversing). We call this alignment scheme the “permitting rewiring and reversing (RR) scheme.” To clarify the effect of RR in the organization of the protein structure, it is beneficial to perform structure alignment using two additional alignment schemes. The first is a type of non-sequential alignment scheme in which structurally equivalent SSEs are allowed to be aligned in different orders in the protein sequences while possessing the same N- to C-terminal direction of the SSEs. We named this alignment scheme the “permitting ReWiring (RW) scheme.” The second is a conventional sequential alignment algorithm in which structurally equivalent regions must be aligned in the same order in the protein sequence. We named this alignment scheme the “SeQuential (SQ) alignment scheme.” Schematic examples of structure alignment using the RW and RR alignment schemes are shown in Figure 1. It would be interesting to use all three alignment schemes for the same dataset and compare their results, as it would provide a better understanding of the physical or geometrical aspects of protein structures, such as the robustness against reversing the orientation of the chain or rewiring the SSEs while maintaining a given spatial arrangement of SSE.

To assess the three different structural alignment schemes, we require a program that can implement all of them. One of the most suitable programs for such an analysis is the non-sequential protein structure alignment program MICAN [26], which we have been developing. MICAN was originally designed to identify the best structural alignment between a protein pair irrespective of chain connectivity. The RW scheme is used in the default setting of the MICAN program, although the search scheme can easily be changed to the RR scheme by specifying the command line option. In a previous paper [26], we provided a detailed description of the algorithm of the RW and RR alignment schemes and presented the results of their benchmark tests. In addition, we recently implemented the SQ search scheme in the MICAN program (the detailed description of the algorithm will be published elsewhere). Accordingly, the current version of the MICAN program can perform structure alignment with any of the three search schemes. In the three search schemes, MICAN optimizes the same scoring function of protein structural similarity using the same optimization procedure, excluding the restrictions imposed in each scheme. This feature makes the program particularly suitable for comparing structure alignments obtained using the three different schemes because the differences in structure alignments arise purely from the differences in the restrictions imposed in each alignment scheme. Another notable feature of MICAN is that it is one of the best programs for reproducing reference alignments created by human experts [26]. For example, the SQ search scheme of MICAN reproduced the reference alignments obtained in a benchmark test set of the MALIDUP [27] and MALISAM [28] databases with accuracies of 89.3% and 79.3%, respectively. The reference alignments in these two databases were carefully inspected and manually curated to ensure good alignment quality by considering geometric similarity and evolutionary and functional relationships. It is widely accepted that these reference alignments are biologically or physically meaningful [29], [30]. Thus, strong agreement with the reference alignments obtained by MICAN suggests that the program provides meaningful structure alignments.

## Results and Discussion

In this paper, we used the TM-score as a metric of structural similarity [31]. Briefly, a TM-score for a pair of protein structures lies in the range [0,1), where a TM-score <0.17 corresponds to a random similarity, and a TM-score  = 1.0 corresponds to identical structures. Statistical analysis revealed that a TM-score ≥0.5 implies that the structures share the same topology [32]. The definition of the TM-score is given in Methods section. To conduct the analysis, we prepared target proteins, which were used as queries to search for similar SSE packing structures. The target proteins were derived from the fold representatives of the SCOP 1.75B database [33], and consist of 1085 structures. The detailed description of the target protein set is given in Methods section. For each target protein, we performed a structural alignments against the remaining 1084 target structures, which we called template structures of the target protein, with the SQ, RW, and RR schemes using the MICAN program and calculated the TM-score(target 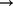 template). Note that throughout this paper, the TM-score always denotes TM-score(target 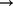 template) opposed to TM-score(template 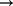 target) (See Methods section).

### How many protein folds share the same SSE packing as at least one other fold?

**Figure 2:**
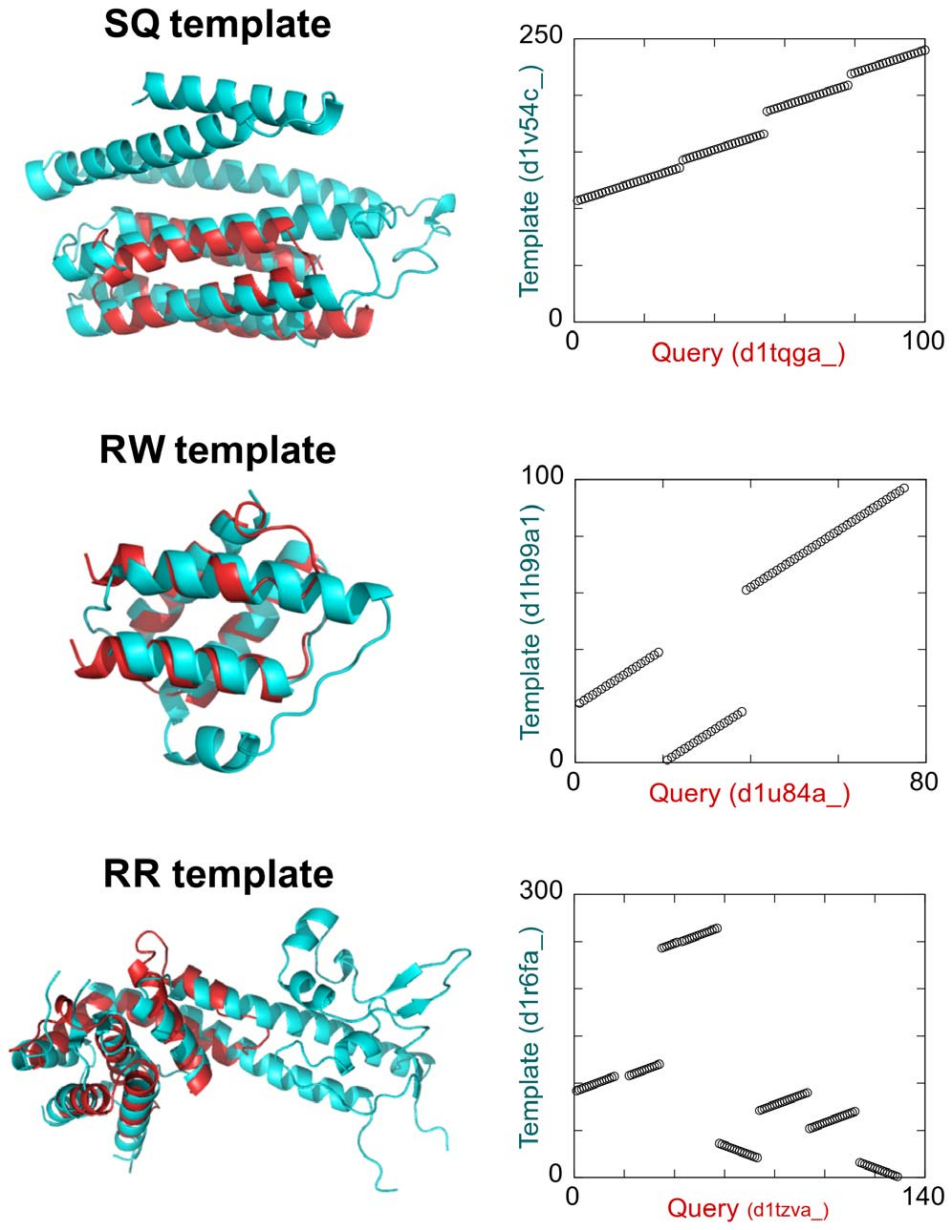
Typical examples of the SQ, RW, and RR templates. The cartoon structures on the left present the superposition structures of a query (red) and the closest structure (cyan) to the query. The figures on the right are alignment plots, on which aligned residue pairs are represented as circles. The horizontal axis of the plot represents the residue number of the query protein, and the vertical axis represents that of the template.

**Figure 3:**
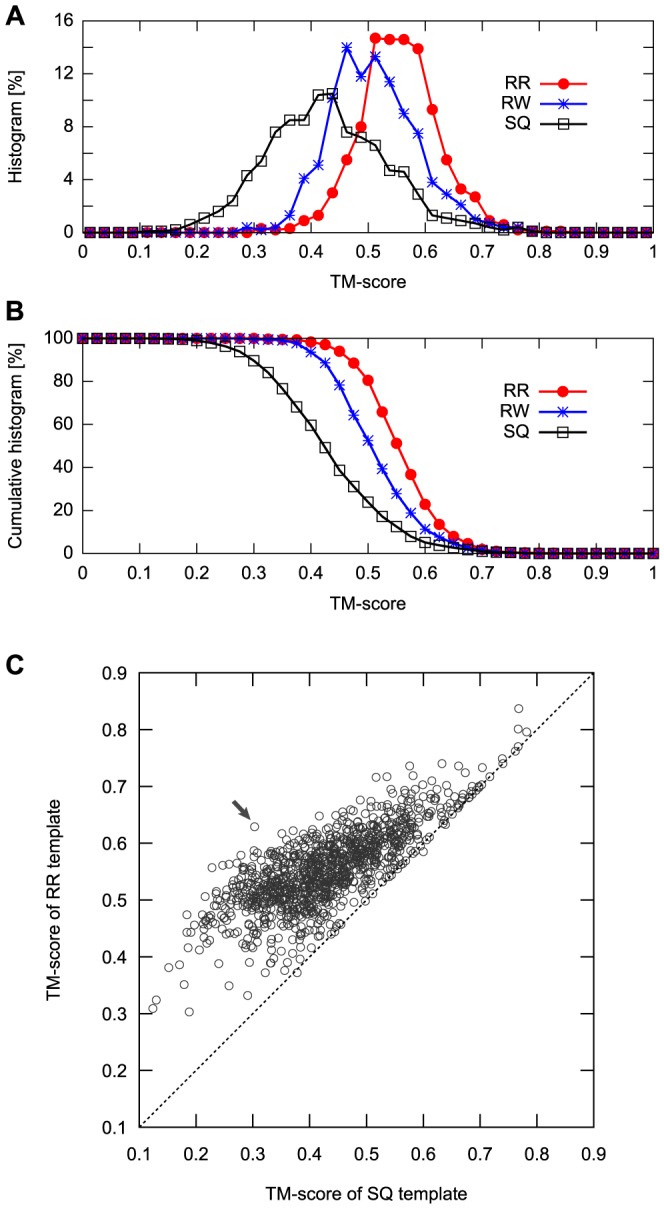
Comparison of the TM-scores among the best SQ, RW, or RR template and the corresponding query. (A) The histograms of the TM-scores of the SQ, RW and, RR templates are represented as black, blue, and red lines, respectively. (B) The cumulative histogram of the target proteins with the TM-scores of SQ, RW, and RR templates that is equal to or greater than the abscissa. (C) The scatter plot of the TM-scores of the SQ template versus RR template. The horizontal axis represents the TM-scores of SQ template, and the vertical axis represents those of the RR template. The black arrow indicates the target displaying the largest difference of TM-score between the SQ and RR templates.

Because the alignment search space of the RR scheme for a given protein pair is much larger than that of the SQ and RW schemes, it is obvious that the largest TM-score obtained by a structure database search for a given query protein structure with the RR method is equal to or larger than those obtained using the SQ and RW methods. However, the difference of these values is not trivial, and we first examined this issue. For each target structure, we identified the closest structure measured by the TM-score using each alignment scheme. The closest structures identified by the SQ, RW, and RR schemes were named as SQ, RW, and RR templates, respectively. Typical examples of the SQ, RW, and RR templates are shown in Figure 2. Figure 3(A) presents the distribution of the TM-scores of the SQ, RW, and RR templates. The result illustrates that the distributions are significantly different from each other; according to the Wilcoxon signed rank test, p-value was smaller than 0.001 for all pairings. The mean TM-scores of the distributions were 0.428, 0.510, and 0.553 for the SQ, RW, and RR templates, respectively. As a TM-score of 0.5 was shown to be a good criterion for assessing structural similarity [32], the RW and RR templates have meaningful similarities to their target structures on an average. Therefore, the results indicate that a large number of protein folds share the same spatial arrangement of SSEs as other folds.

To determine how many protein folds share the same SSE packing as at least one other fold, we computed the cumulative histogram of the target proteins with equal or greater TM-scores of the SQ, RW, and RR templates than the abscissa (Figure 3(B)). Using a TM-score of 0.5 as the cutoff for structural similarity, the percentage of the targets with similar SQ, RW, and RR templates were 23.9%, 52.5%, and 80.5%, respectively. This result suggests that approximately 80% of protein folds share the same SSE packing arrangement as at least one other fold.

To elucidate what types of query proteins display large differences in the TM-scores with changes in the alignment scheme, we drew a scatter plot of the TM-score of the SQ templates versus that of the RR templates, as shown in Figure 3(C). A large difference was observed for the targets with SQ template TM-scores smaller than 0.6. In particular, for the targets with SQ template TM-scores of 0.3–0.4, the difference was significant, and their RR templates display moderate structural similarity (TM-score ∼0.5) with the target structure. This result suggests that many of the topologically different (TM-score ∼0.3) protein pairs share the same spatial arrangement of SSEs. In contrast, almost no difference was observed for the targets with high-quality SQ templates (TM-score ≥0.6). These observations suggest that topologically similar protein pairs (TM-score ∼0.6) do not exhibit significantly greater structural similarity even if the chain connectivity and direction of SSEs are ignored. Note that most of the targets with high-quality SQ templates (TM-score ≥0.6) are small proteins and their templates are much larger than the target; the average protein size of these targets is 98.3 residues and that of their SQ templates is 278.2 residues. Thus, high-quality SQ templates were found in other folds primarily because of the Russian doll effect, i.e., smaller protein structures are contained within larger structures [34].

**Figure 4:**
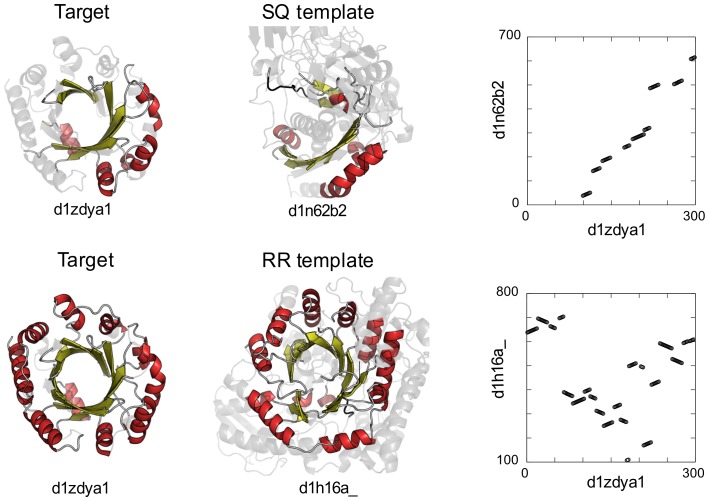
The SQ and RR templates of aromatic prenyltransferase. The cartoon figures on the left represent the structure of aromatic prenyltransferase, which exhibited the largest difference in TM-score between the RR and SQ templates, and those in the middle present the SQ and RR template structures. In these figures, only structurally aligned regions are highlighted and colored. The graphs on the right are the alignments plots between the target and template.

The target protein with the largest difference in TM-score between the RR and SQ templates is aromatic prenyltransferase (SCOP ID: d1zdya1). The TM-score of the RR template is 0.629 and that of the SQ template is only 0.303. The corresponding data are indicated by arrows in Figure 3(C). The structure of this target is a PT-barrel fold (Figure 4), which consists of a 10-stranded up-down *β*-barrel and 9 helices that surround the *β*-barrel. As shown in Figure 4, the SQ template structure (SCOP ID: d1n62b2) presents partial structure similarity to the target; the structurally aligned region covers only 9 (6 strands and 3 helices) of 19 SSEs. In contrast, the structure of the RR template (SCOP ID: d1h16a_) exhibits overall structural similarity in a non-sequential manner, and the structurally aligned regions cover all 19 SSEs of the target structure. The structure alignment consists of several non-sequential fragments, 8 of which are aligned in the opposite direction of the SSEs. This example emphasizes the importance of both rewiring and reversing of SSEs for identifying the same SSE packing structures.

### The target size dependence of the probability of finding a protein fold that shares the same spatial arrangement of SSEs

As previously observed, the target proteins with high-quality SQ templates are small proteins, which suggests that the probability of a target protein having similar structures in other folds strongly depends on the target protein size. Here we investigated the protein size dependence of the percentage of target proteins with at least one similar structure in other folds in a more quantitative manner.

**Figure 5:**
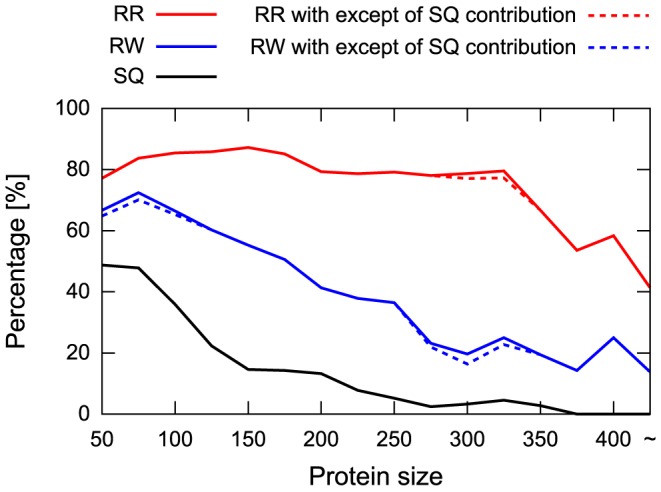
Analysis of the target size dependence. The percentage of target proteins with a TM-score of the SQ, RW, and RR templates equal to or greater than 0.5 as a function of the target protein size is presented. The horizontal axis represents the sequence length of the target proteins. The vertical axis represents the percentage of the target proteins with the TM-scores of SQ, RW, and RR templates equal to or greater than 0.5 for a given target size. The lines are colored in black, blue, and red for the SQ, RW, and RR templates, respectively. The dotted lines correspond to the data in which the contributions of protein folds that exhibit topological similarity to the target proteins are excluded.

Figure 5 shows the percentage of target proteins with SQ, RW, and RR template TM-scores equal to or greater than 0.5 as a function of a given target size. The results using other thresholds, which show qualitatively similar behaviors, are also shown in Figure S1. The most remarkable finding is that the probability of finding a similar structure (TM-score ≥0.5) using the RR scheme is high over the wide range of target size and robust against the target protein size, whereas the probability of finding a good SQ and RW template rapidly decreases with an increase in protein size. For instance, for a protein size of 150 residues, which is the average chain length of the protein domain defined by the SCOP database, as many as 87.2% of the target proteins have similar (TM-score ≥0.5) RR templates in other folds, whereas only 14.6% and 55.3% of them have good SQ and RW templates, respectively. The difference becomes more significant as the target protein size increases. Even for a protein size of 300 residues, 78.7% of the target proteins have similar RR templates, whereas only 3.3% and 29.7% of the target proteins have good SQ and RW templates, respectively. These results suggest that considering the reverse orientation of protein chains has a significant effect on identifying structures with the same spatial arrangement of SSEs.

### The contribution of topologically similar structures assigned to the different SCOP folds

One of the aims of this study was to estimate the number of protein folds that share the same spatial arrangement of SSEs with topologically different folds. So far, we have assumed that all of the SCOP fold representatives have different topologies from each other. However, as shown in Figure 3, some target structures have high-quality SQ templates (TM-score ∼0.6), suggesting that some topologically similar structures have different SCOP folds. In fact, such redundancy of the SCOP fold assignment has been observed by many groups [7], [8], [35], [36]. Possible reasons for this redundancy are the Russian doll effect and the classification criteria of the SCOP database, which are based on protein geometry as well as evolutionary and functional considerations. Such redundancy may result in overestimation of the number of protein folds that share the same spatial arrangement of SSEs with topologically different folds because it allows the possibility that the RW or RR template exhibits topological similarity to its target with high TM-scores (the RR templates with high TM-scores on the diagonal line of the scatter plot in Figure 3 are likely to be such templates).

Here we reassessed the number of protein folds that share the same spatial arrangement of SSEs with at least one topologically different fold, eliminating the contribution of protein folds that exhibit topological similarity to the target proteins, using a TM-score threshold of 0.5 (see Methods section for details). As a result, we found that the percentages of such folds were 51.7% and 80.4% for the RW and RR schemes, respectively. These values are similar to those obtained in the previous section (52.5% and 80.5% for the RW and RR schemes, respectively), suggesting that our estimation presented in the previous sections is robust against the fold assignment. We also examined the effect of the redundancy of the SCOP fold assignment on the target size dependence of the probability of finding similar SSE packing structures and confirmed that it had little influence on the results (refer to the dotted lines shown in Figure 5).

### A small minority of protein folds share the same spatial arrangement of SSEs with a large number of other folds

So far, we have focused on whether a given target protein has at least one similar packing structure in other folds. Next, we examine how many structural neighbors a given target protein has in other folds. For this purpose, we defined the structural neighbor 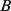 for a given target protein 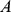 as the fold representative that has a TM-score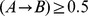. For each target, we identified the structural neighbors using the SQ, RW, and RR schemes and counted the number of the structural neighbors using the three schemes, which are referred to as 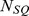, 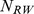 and 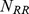, respectively. Note that because the RR and RW schemes allow a larger search space than the SQ scheme, it necessarily holds that 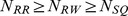.

To obtain an overview of the number of structural neighbors according to the alignment scheme, we first calculated the average values of 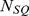, 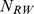, and 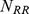 per target protein. The average number of structural neighbors significantly increased as the restriction of the alignment search space was relaxed from the SQ scheme to the RW scheme and from the RW scheme to the RR scheme. The resulting values were 2.43, 7.78, and 26.44 for 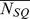, 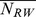, and 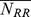, respectively, with the overline denoting average values.

Note that 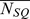, which reflects the number of topologically similar structures in other SCOP folds, is not negligible. As described in the previous sections, a non-zero value of 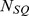 results in overestimation of the values of 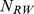 and 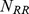, which reflects the number of non-sequentially similar but topologically dissimilar structural neighbors. To avoid such overestimations, we hereafter focus on 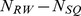 and 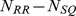, rather than 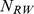 and 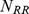.

**Figure 6:**
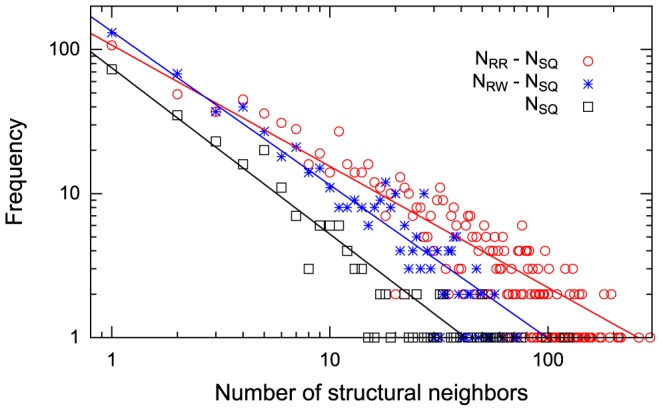
Distributions of the number of structural neighbors. The distributions identified by the SQ, RW, and RR schemes are shown in the double logarithmic plot. The contribution of the number of topologically similar structures is excluded for 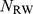 and 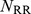. The red, blue, and black points represent the data from the SQ, RW, and RR schemes, respectively. These distributions are well fitted to the power-law model, as illustrated by the solid lines.

Figure 6 shows the distributions of the number of structural neighbors. All three distributions are strongly biased, and each distribution follows a power-law method with statistical significance (p-value 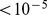), implying that a small minority of protein folds have a large number of structural neighbors and a large majority have a few neighbors. For example, 80% of protein folds have an 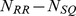 value of less than 30, whereas only 5% have a value larger than 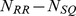 of 100. The largest values of 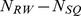 and 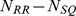 were 73 and 294, respectively. The large difference between 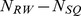 and 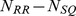 implies that many protein pairs that share the same spatial arrangement of SSEs include structurally equivalent SSE pairs having opposite chain directions.

**Figure 7:**
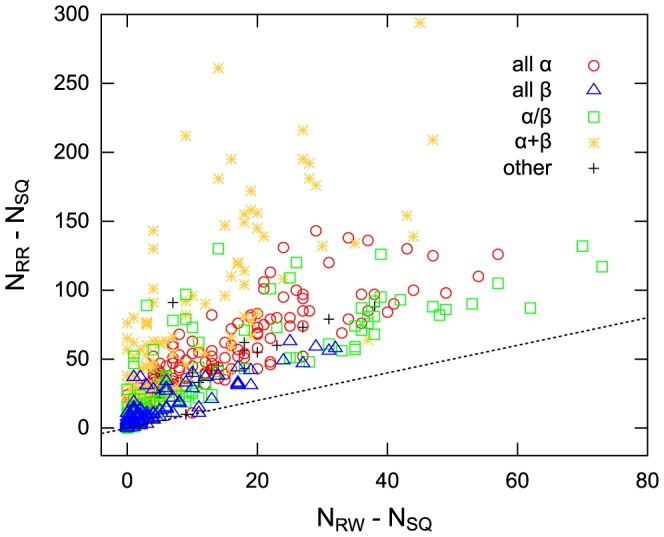
Scatter plots showing 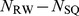 versus 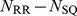 and 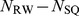 versus 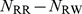 for all fold representatives. The colors of the plots represent the SCOP classes as follows: all-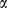 (red), all-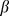 (blue), 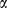/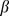 (green), 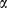
+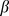 (yellow), and others (black). The break line represents the equation 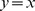. (A) and (B) respectively represent the scatter plots showing 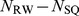 versus 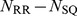 and 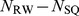 versus 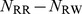.

The choice of the structure alignment scheme dramatically changes both the number of structural neighbors and the ranking of protein folds in terms of the number of structural neighbors. Figure 7(A) presents a scatter plot of 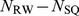 versus 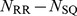, in which each point represents a target protein, which is colored according to its SCOP class as follows: all-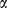 (red), all-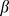 (blue), 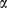/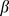 (green), 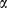
+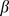 (yellow), and others (black). As the figure indicates, the protein fold with the largest 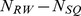 value does not correspond to that with the largest 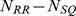 value. Moreover, the SCOP classes of these two protein folds are different. The former belongs to the 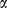/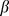 class, whereas the latter belongs to the 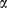
+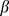 class. In addition to the fold with the largest structural neighbors, the ranking of several other folds in terms of the number of structural neighbors, depending on the alignment scheme. In particular, the ranking and number of structural neighbors of some folds belonging to the 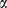
+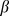 class change dramatically based on the alignment scheme; some 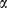
+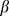 protein folds with relatively small 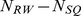 value (10–30) have extremely large 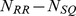 value (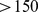). These results suggest that the number of structural neighbors, as well as the ranking, is strongly affected by the choice of the structure alignment scheme.

It is instructive to examine how the number of structural neighbors increases as the alignment scheme changes from SQ to RW or from RW to RR. For this purpose, we compare 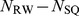 with 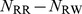. Figure 7(B) shows a scatter plot of 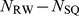 versus 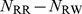, in which the coloring scheme is the same as that in Figure 7(A). In the scatter plot, points above the diagonal line indicate that the increase in the number of structural neighbors by ignoring the chain direction is larger than that by ignoring only the connectivity of SSEs and points below the diagonal line indicate the opposite situation. As the figure indicates, for most target proteins, 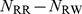 is larger than 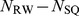, implying that permitting reversal of the chain direction has a stronger influence on increasing the number of structural neighbors than just ignoring the connectivity of SSEs.

One of the well-known naturally occurring phenomena that show a non-sequential structural relationship is a circular permutation (CP) [37]–[40]. CP is a protein structural rearrangement phenomenon in which the connectivity of a protein is altered by connecting the N- and C-termini of a protein with a peptide linker and creating new termini elsewhere. It is interesting to investigate the prevalence of CPs in our dataset. Here we assessed the frequency with which CP relationships were observed in protein pairs with significant structural similarity (TM-score ≥0.5) identified by the RW scheme. We found that there are 8424 protein pairs with significant structural similarity identified by the RW scheme and that 1645 (19.5%) of them show a CP relationship, according to the procedure of Abyzov and Ilyin [12]. The observed frequency of CP is significantly higher than its random expectation; given that the average number of SSEs of the target proteins considered here is approximately 7 (6.76), the number of possible connectivity patterns is estimated as 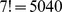 and there are 6 alternative CP variants for a given order of SSEs. Thus, the naive probability of the occurrence of CP is calculated as 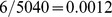, which is far smaller than the value we observed (0.195). One possible explanation for the large difference in the occurrence of CP is physical limitations for some types of structures [41]. For instance, protein topologies with knots or loop crossings are severely restricted and rarely observed in natural proteins [42]–[44]. Such topological restrictions should reduce the number of possible connectivities. On the other hand, CP is one of the simplest ways to change the topology of proteins, if the N- and C-termini are close to each other. Thus, the observed probability of the occurrence of CP is expected to be much higher than its random expectation. A more detailed analysis would be interesting and important but is left for a future study.

### What types of spatial arrangements of SSEs are frequently observed across fold space?

**Figure 8:**
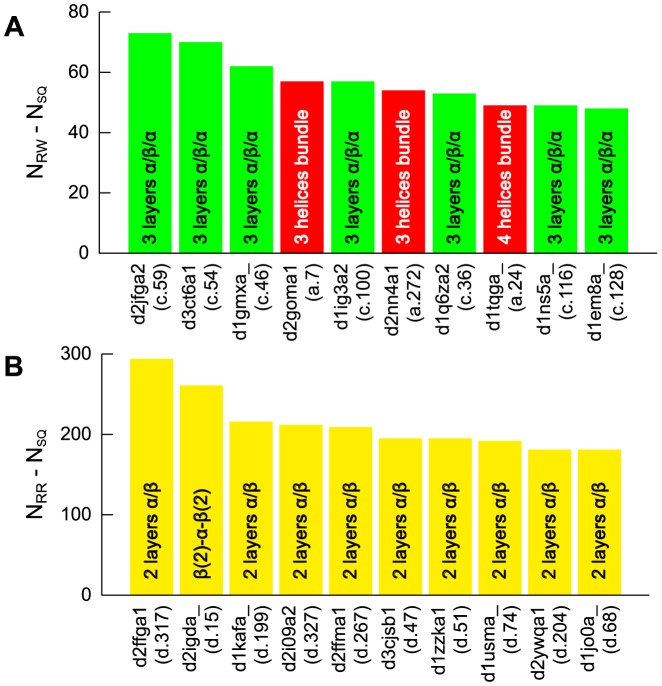
The top 10 SCOP folds having the largest numbers of structural neighbors identified by the RW (A) and RR (B) schemes. Each bar represents a fold representative, and it is colored by SCOP class as follows: all-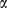 (red), all-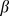 (blue), 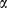/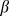 (green), 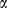
+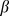 (yellow), and others (black). The height of the bar represents 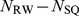 or 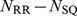. The target proteins are ordered by their values of 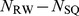 or 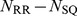. For each target, the SCOP ID and SCOP fold ID are given under the bar. Short descriptions of each fold are also given in the bars.

We investigated the types of protein folds having large numbers of structural neighbors depending on the alignment scheme. Figure 8 presents top 10 protein folds with the largest number of structural neighbors as identified by the RW and RR schemes.

**Figure 9:**
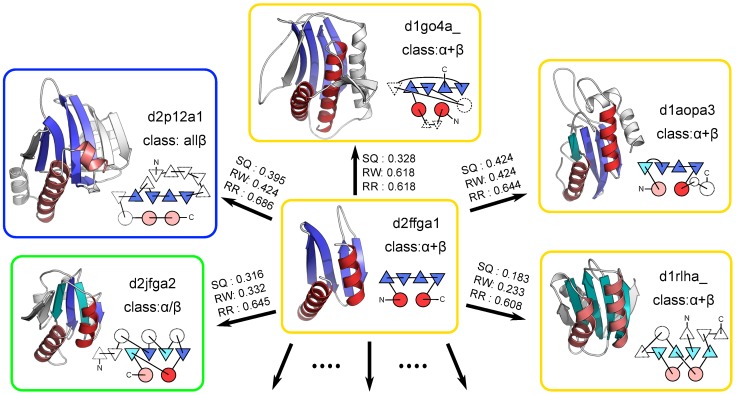
Structures of the target d2ffga1 and its structural neighbors. The cartoon representation of the protein structure possessing the most frequently observed spatial arrangement of SSEs (d2ffga1) and five examples of its structural neighbors (d1go4a_, d1aopa3, dlrlha_, d2jfga2, and d2p12a1) are presented. This spatial arrangement of SSEs consists of four strands and two helices, which are highlighted by colors in each structure. In the structure of d2ffga1, the strands and helices are highlighted in blue and red, respectively. In the other structures, the colors of the strands and helices with the same chain direction as those in d2ffga1 are identical to those in d2ffga1. The helices and reverse strands with opposing directions are colored in salmon and cyan, respectively. The connectivity diagrams are also shown near the cartoon representations. The color scheme is the same as those for the cartoon representations. The TM-score(d2ffga1 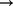 example) calculated by the SQ, RW, and RR schemes is also shown.

As observed in Figure 8(A), most of the top 10 protein folds (7 of 10) according to the RW scheme belong to the 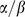 class, and they all have the 3-layer 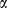/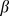/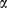 packing arrangement. The protein fold with the largest 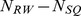 value is the MurD-like peptide ligase fold. The representative protein of the fold is D-glutamate ligase MurD (SCOP ID: d2jfga2), and its structure and topological cartoon are shown in Figure 9. As described previously, 73 protein folds have the spatial arrangement of SSEs adopted by d2jfga2. Most of them (63 of 73) belong to the 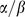 class, indicating that most of the fold pairs that share same spatial arrangement of SSEs are confined within the same SCOP class, as long as the RW scheme is used.

Unlike the case of the RW alignment scheme, the RR alignment scheme strongly prefers the 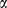
+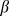 class. As shown in Figure 8(B), all of the top 10 SCOP folds ranked by 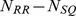 value belong to the 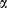
+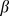 class, and 9 of them have the 2-layer 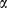/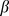 packing arrangement. A typical example of the 2-layer 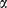/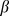 packing arrangement is the YkuJ-like fold, which has the largest 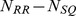 value. The representative protein with the YkuJ-like fold is the hypothetical protein YkuJ (SCOP ID: d2ffga1), and its structure is shown in Figure 9. As the spatial arrangement of SSEs adopted by the YkuJ-like fold becomes most frequently observed across the fold space only if the reverse alignment is allowed, many structural neighbors of the YkuJ-like fold have at least one SSE that has the reverse orientation to that of the structurally equivalent SSE of the YkuJ-like fold. In addition, its spatial arrangement may be one of the most physically or geometrically preferred arrangement independent of the chain direction of the SSEs.

What types of protein folds are the structural neighbors of the YkuJ-like fold? As previously mentioned, when the RR scheme is used, the YkuJ-like fold has 294 structure neighbors. Analyzing the structural classes included in the 294 structural neighbors, we found that 0, 12, 122, 124, and 36 of the neighbors belong to the all-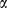, all-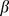, 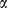/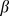, 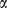
+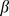, and the other classes, respectively. This observation suggests that the structural neighbors of the YkuJ-like fold include a wide variety of protein folds across different classes of the SCOP database. The number of structural neighbors of the YkuJ-like fold identified by the RW scheme was 45, which was much smaller than that identified by the RR scheme. This result indicates that many structural neighbors of the YkuJ-like fold identified by the RR scheme have at least one structurally equivalent SSE having the opposite chain direction of those of the YkuJ-like fold.

Five examples of the structural neighbors of the YkuJ-like fold are shown in Figure 9. The first example is d1go4a_. This example belongs to the same SCOP class (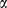
+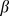) as the YkuJ-like fold. Although their SSE connectivities are different, all of the structurally equivalent SSEs have the same direction. Accordingly, the TM-score(d2ffga1 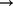 d1go4a_) obtained by the RW scheme and that obtained by the RR scheme were identical. The second example is d1aopa3, which is also classified in the 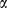
+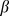 class. The notable feature of this pair is that 2 of 6 structurally equivalent SSEs have opposing directions. The third example is d1rlha_. This example is an extreme case in that all of the structurally equivalent SSEs have an opposing direction to the target. The other two examples are d2jfga2 and d2p12a1, which are illustrated to show that the structural neighbors can disperse across different SCOP classes; d2jfga2 belongs to the 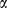/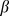 class, and d2p12a1 belongs to the all-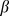 class. These examples emphasize that structural neighbors spread to a wide variety of protein folds even across the SCOP classes and that the frequent appearance of opposing directions among structurally equivalent SSEs.

A similar but different analysis was performed by Harrison *et al.* [21]. They calculated “gregariousness,” which measures how many other folds have a significant structural overlap with a given fold regardless of the chain connectivity. Their work shares certain similarities with ours, i.e., both of them used an RW-like scheme. However, their analysis differs from ours because they did not consider the SQ and RR schemes.

### Many structural neighbors are found in a wide variety of folds even across the SCOP classes

In the previous section, we identified the protein fold that adopts the most frequently observed spatial arrangement of SSEs and briefly discussed the protein folds that share the same spatial arrangement of SSEs with the fold. In this section, we expanded this analysis to include all fold representatives and investigated the protein fold universe in terms of how the different protein folds share the same spatial arrangement of SSEs using the SQ, RW, and RR alignment schemes. Comparing the comprehensive view of protein space obtained using the three alignment schemes will provide a better understanding of the role of rewiring the SSEs, as well as reversing their chain direction, in connecting different folds in the universe of protein folds. For this, we employed a directed graph representation of the protein fold universe, in which a node of the graph represents a fold representative. The directed edge between 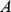 and 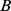 is created from 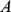 to 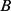, if the TM-score(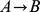) is larger than 0.5. Because the graphical representation of protein structures is useful for investigating the protein structure universe, it has been widely used by many researches [7], [45]–[47].

**Figure 10:**
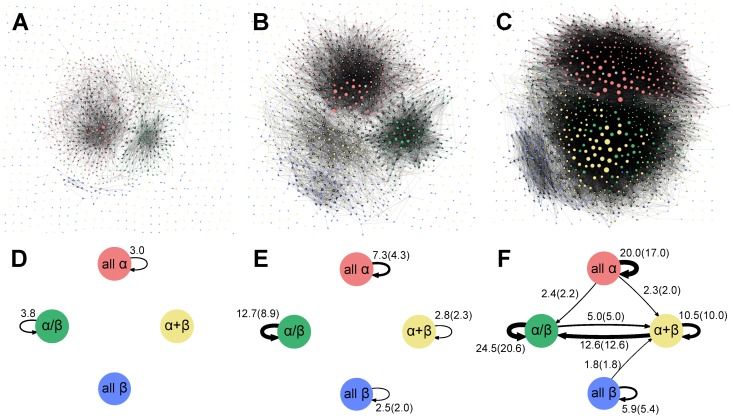
Graphical representation of the protein fold universe. (A)–(C) The detailed graphical representations of the protein fold universe connected by the SQ (A), RW (B), and RR (C) schemes. In these networks, protein folds are represented by nodes and connected by the directed edge. The directed edge between 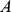 and 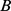 is created from 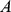 to 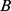 if the TM-score(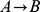) is larger than 0.5. The node size is proportional to the out-degree of the node. Nodes are colored according to their SCOP class as follows: all-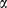 (red), all-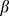 (blue), 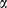/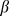 (green), 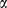
+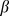 (yellow), and others (black). (D)–(F) The simplified networks of the protein fold universe based on the SQ (D), RW (E) and RR (F) networks. Each node represents all-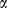, all-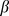, 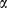/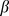, and 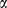
+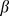 classes defined in the SCOP database. The directed edge is drawn if 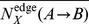 is larger than 1.0, where 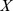 represents the alignment scheme. The numerical value of 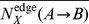 is shown near the edge. The numerical values shown in parentheses in Figure 9(E) and (F) are 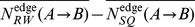 and 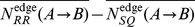, respectively. The width of the arrows indicates the numerical value of 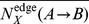.

Figures 10(A), (B), and (C) show the graph representations of the protein fold universe connected by the SQ, RW, and RR schemes. We refer to the graph connected by the SQ, RW, and RR schemes as the SQ, RW, and RR networks, respectively. The protein folds are colored according to their SCOP class as follows: all-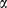 (red), all-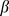 (blue), 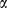/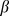 (green), 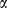
+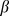 (yellow), and other classes (gray). The overall impressions of the figures are as follows. (i) The SQ network is mostly composed of isolated fold islands, excluding some all-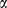 and 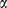/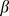 folds. The exceptions of all-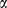 folds can be explained by the Russian doll effect. Finding many edges within the 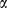/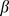 class is consistent with the indication that topologically similar Rossmann-like structures are categorized into approximately 80 different SCOP folds based on their function [36]. (ii) In the RW network, we can clearly observe four clusters. Interestingly, these clustered approximately correspond to the all-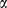, all-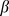, 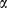/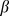 and 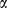
+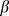 classes defined in the SCOP database, although the SCOP class assignment of the folds was not used in constructing the graph. This observation suggests that the role of permitting SSE rewiring in connecting different folds is linking many nodes within the same SCOP class. In other words, many protein pairs within the same SCOP class share the same spatial arrangement of SSEs with different topologies while preserving the N- to C-terminal direction of SSEs. (iii) In the RR network, we can observe three clusters. Two of them roughly correspond to the all-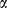 and all-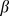 SCOP classes. In the third cluster, unlike in the RW network, protein folds belonging to the 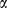/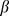 and 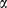
+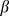 SCOP classes are well connected, forming a large cluster. Compared with the RW network, the boundaries between the clusters are ambiguous, suggesting that there are considerable edges across different classes. This observation indicates that the primary role of permitting reversal of the chain direction of SSEs is connecting many nodes across different SCOP classes. It also implies that many fold pairs belonging to the different SCOP classes share the same spatial arrangement of SSEs.

To understand the networks in an intuitive manner and characterize them quantitatively, we constructed simplified networks of the protein fold universe based on the SQ, RW, and RR networks (Figures 10(D), (E), and (F)). The simplified network comprised only four nodes, which represent the all-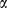, all-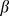, 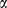/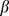 and 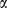
+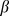 classes defined in the SCOP database. The directed edge was created on the basis of the average number of edges across or within the SCOP classes in the SQ, RW, and RR networks. The average number of edges that connect from class 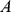 to class 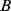 in the 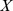 (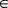 SQ, RW, RR) network is defined as 
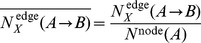
(1)where 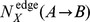 is the total number of edges that connect from nodes belonging to class 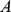 to those belonging to class 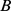 in the 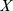 network and 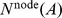 is a number of nodes belonging to class 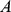. If 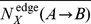 is larger than 1.0, then we drew the directed edges between the nodes and presented its numerical value near the edge in the simplified networks. To clarify how large the contribution of topologically similar fold pairs categorized into different SCOP folds is, we calculated 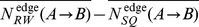 and 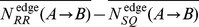, and presented these numbers in parentheses in Figures 10(E) and (F).

In the simplified SQ network, consistent with the overall impression, there are no inter- and intra-class connections excluding the intra-class connections of the all-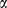 and 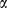/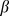 classes. To check the consistency with other methods, we constructed the network using TM-align [48] and HHsearch [49], and confirmed that the isolated picture of the SQ network produced by MICAN is reasonable (See Text S1, Figure S3 and S4). Compared with the simplified SQ network, the simplified RW network has significantly more intra-class connections; the increases in the average number of intra-class connections of the all-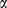, all-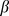, 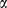/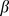 and 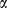
+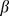 classes are 4.3, 2.0, 8.9, and 2.3, respectively. Conversely, the average number of inter-class connections is still small in the simplified RW network, with all values being less than 1.0. Thus, no edge was drawn between any node pairs. These observations indicate that for a given protein fold, on average, we can find 2–9 other folds that have the same spatial arrangement of SSEs preserving the same chain direction within the same SCOP class. The simplified RR network is largely different from both the simplified SQ and RW networks. The most notable feature of this network is that there are inter-class connections that reflect a large average number of inter-class connections in the RR network. In particular, the connection between the 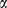/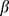 and 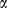
+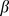 classes is extremely strong; the average number of edges that connect from 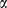
+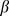 class to 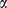/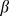 class in the RR network is 12.6. Other inter-class connections are also significant, although not as numerous as those between the 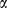/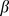 and 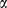
+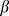 classes. It should be noted that both the inter- and intra-class connections are extremely strong in the simplified RR network; the average numbers of edges within the class in the RR network are much larger than those in both the SQ and RW networks. The results indicate that for a given protein fold, we can find many other folds that have the same spatial arrangement of SSEs in a wide variety of protein folds across different classes as well as within the same class of the SCOP database if we ignore both the connectivity and the N- to C-terminal direction of the SSEs.

### Implications for protein structure prediction

The results reported in this study are important for protein structure prediction. As mentioned previously, 80% of protein folds share the same SSE packing arrangement with at least one other fold. This result implies that for approximately 80% of “new fold” targets, we can generate good models from “old folds” via multiple loop permutations [16]–[18] and reversing the chain direction. Because the primary obstacle to *de novo* protein structure prediction is thought to be conformational sampling rather than inaccuracy of energy functions [50], developing a method for generating structures via the rewiring/reversing technique may open a new path for *de novo* protein structure prediction.

To demonstrate the potential usage of the RW and RR templates for targets for which no suitable template can be identified even using the state-of-the-art threading programs, we performed additional calculations using a different target/template set. Here we chose the template free modeling (FM) targets in the 10th Critical Assessment of Structure Prediction techniques (CASP10) experiment as another target set. The FM targets are proteins for which no suitable template can be identified. Although the structures of the FM targets are not always new folds, they are sometimes regarded as new-fold targets [17]. For the 29 FM targets in the CASP10 experiment, we identified the closest templates to the native structure with the SQ, RW, and RR search schemes from the PDB snapshot from 1st May 2012, when the first CASP10 target was released.

**Figure 11:**
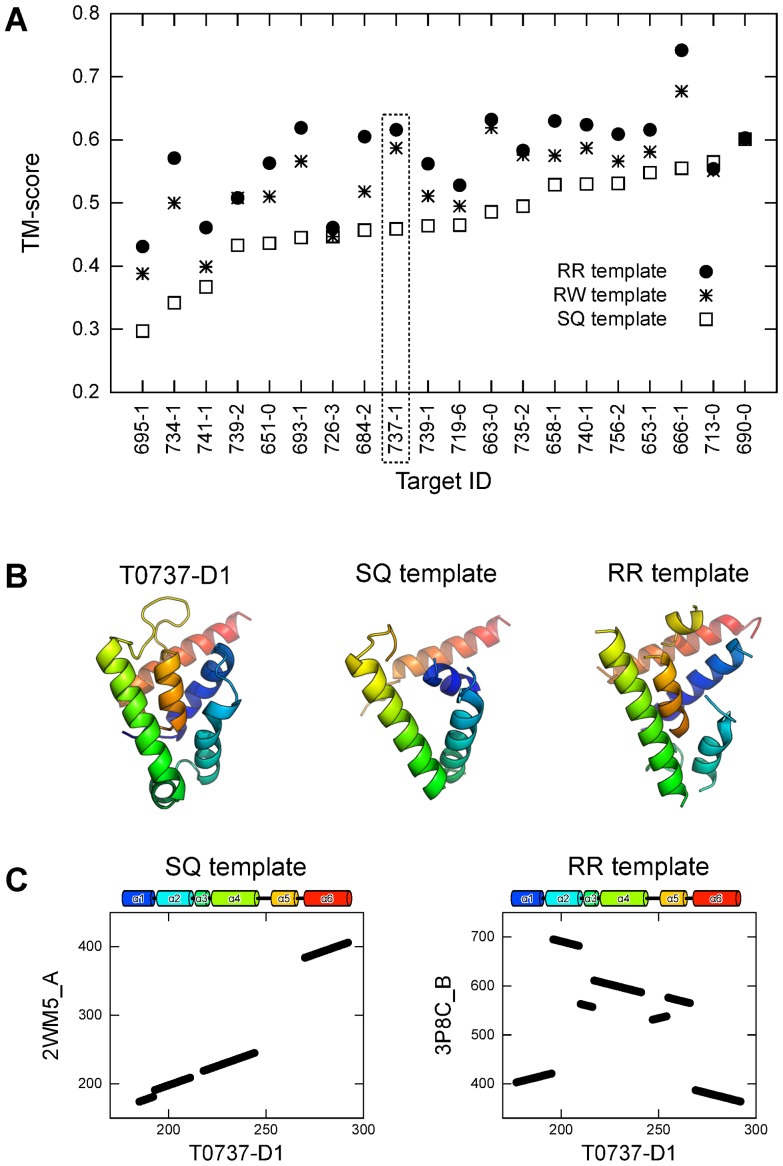
SQ, RW, and RR templates for FM targets in CASP10. (A) TM-scores of the SQ, RW, and RR templates identified from the PDB snapshot for all FM targets in CASP10. The target domains are ordered according to the TM-scores of their SQ templates. Open squares, asterisks, and filled circles represent TM-scores of SQ, RW, and RR templates, respectively. (B) The native (3td7), the SQ template (PDB id: 2wm5), and the RW template (3p8c) structure of the target T0737-1. For the template structures, only the aligned residues are shown as cartoon models. (C) The alignment plots between the native and the SQ template (left) and between the native and the RR template (right).

Figure 11(A) shows the TM-scores of the SQ, RW, and RR templates identified for each target. The targets are ordered according to the TM-scores of their SQ templates. Consistent with the previous observation, for most FM targets, TM-scores of the RR and RW templates are significantly larger than those of the SQ templates. The percentages of the FM targets that have at least one good template (TM-score ≥0.5) in the PDB snapshot are also significantly different among the three: the percentages of SQ, RW, and RR templates are 35.0%, 80.0%, and 85.0%, respectively. These results show the advantage of the rewiring and reversing technique in creating high-quality structure models for the FM targets.

T0737-D1 is an interesting example showing large differences in the TM-score between the SQ and RR templates. Figure 11(B) shows structures of the native, SQ, and RR templates of the target. The SQ template structure (PDB ID: 2wm5) presents partial structural similarity to the target; the structurally aligned region covers four of six helices. In contrast, the structure of the RR template (PDB ID: 3p8c) exhibits overall structural similarity; the structurally aligned regions cover all six helices of the target structure. Interestingly, four structurally equivalent helices of the RR template have the opposite chain direction of SSEs of the native structure, as shown in Figure 11(C). The TM-scores of the SQ and RR templates are 0.459 and 0.616, respectively. These results confirm the potential utility of the rewiring and reversing technique in structure modeling.

As mentioned above, structure modeling by rewiring and reversing has great potential to predict tertiary structures for targets for which no suitable template can be identified even by the use of state-of-the-art threading programs. Unfortunately, such an approach for structure prediction is not straightforward at present. One of the most serious problems is that a connectivity- and direction- independent mapping of the sequence onto the structure has not been developed. Developing such an algorithm is important but is left for future studies.

## Conclusion

In summary, we clarified the frequency with which protein folds share the same SSE packing arrangement with other folds, the types of spatial arrangement of SSEs that are frequently observed across different folds, and the diversity of protein folds that share the same spatial arrangement of SSEs with a given fold, using the protein structure alignment program MICAN. For these issues, we investigated whether the results are affected by the use of three different alignment schemes: the SQ, RW, and RR schemes.

By performing structural similarity searches of each SCOP fold representative against the other folds using the SQ, RW, and RR schemes and a TM-score cutoff of 0.5, the percentages of the fold representatives that have at least one similar SQ, RW, and RR template in the other folds were determined to be 23.9%, 52.5%, and 80.5%, respectively. The result indicates that approximately 80% of protein folds share the same spatial arrangement of SSEs as other folds and that the effects of rewiring and reversing of SSEs in structure comparisons is significant.

The most frequently observed spatial arrangement of SSEs in our analysis was the 2-layer 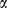/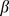 packing arrangement adopted by the YkuJ-like fold. This spatial arrangement of SSEs is present in as many as 294 of 1085 folds. For these 294 folds, SSEs with structural equivalence to those of the YkuJ-like fold frequently had the opposite chain direction, suggesting the robustness against reversal of the chain direction of SSEs for maintaining the spatial arrangement of SSEs.

The graphical representation of the protein universe revealed that if both RR the chain direction of the SSEs are allowed in structure alignment, then protein folds sharing the same spatial arrangement of SSEs with a given fold include a wide variety of protein folds across the SCOP classes. In contrast, if reversing the chain direction of the SSEs is prohibited, most of the fold pairs that share same spatial arrangement of SSEs are found within the same SCOP class. Furthermore, if sequential structure alignment is used, most of the folds do not share the same spatial arrangement of SSEs with any other folds, excluding all-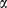 and 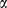/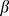 proteins. These results indicate that rewiring connects different folds within the same SCOP class in the universe of protein folds and that reversing the direction of SSEs connects different folds across the different SCOP class.

## Methods

### A metric of structural similarity

We used the TM-score as a metric of structural similarity [31]. The TM-score of the structure of template protein 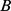 with respect to the target protein 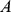 is 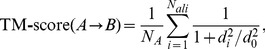
(2)where 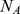 is the length of the target protein 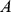, 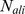 is the number of aligned residues, 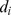 is the distance between the 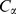 atom of the 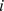-th pair of aligned residues and 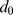 is a scale to normalize the match difference defined as 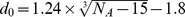. This formula was introduced to eliminate the inherent protein size dependence of the score function. The value of the TM-score lies within 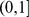. Statistical analysis revealed that a TM-score 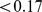 corresponds to a random similarity, whereas a TM-score 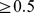 implies that the structures share the same topology [32]. Note that the TM-score is non-symmetric for protein pairs with different lengths: TM-score(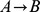) 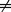 TM-score(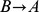). For a given protein pair 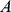 and 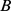, we calculated the TM-score
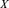
(A 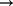 B), where TM-score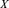 is the TM-score calculated with the 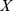 (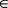 SQ, RW, RR) scheme, using a program MICAN [26].

Theoretically, it necessarily holds that TM-score
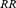
(A 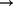 B) 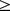 TM-score
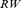
(A 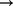 B) 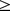 TM-score
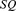
(A 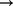 B) because the RR (RW) scheme allows a larger alignment search space than the RW (SQ) scheme. In reality, however, it has been observed that, except in extremely rare cases, TM-score
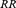
(A 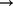 B) is slightly smaller than TM-score
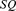
(A 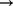 B) for some pairs by MICAN calculation, as shown in Figure 3(C). This is because the algorithm of the SQ, RW, and RR schemes for optimizing the TM-score implemented in the MICAN program is heuristic rather than an exact algorithm [26]. We examined the number of protein pairs that have negative values of (TM-score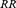 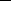 TM-score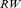), (TM-score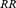 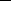 TM-score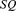), or (TM-score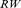 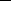 TM-score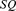), and found that these pairs are quite rare (on average, 1.3%), suggesting that artifacts introduced by the search heuristics are negligible.

### The threshold for sharing the same spatial arrangement of SSEs

Do meaningful TM-score thresholds of the RW and RR schemes for sharing the same spatial arrangement of SSEs significantly differ from that of the SQ scheme? It is widely accepted that a TM-score ≥0.5 generally corresponds to the same fold [17], [51], [52]. Given that protein pairs sharing the same fold necessarily have the same spatial arrangement of SSEs [1], a TM-score of 0.5 seems to be a suitable criterion for sharing the same spatial arrangement of SSEs, even if the RR or RW scheme is used. However, because it holds that TM-score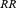 ≥ TM-score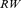 ≥ TM-score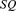, it is instructive to examine how different these values are for protein pairs sharing the same fold.

**Figure 12:**
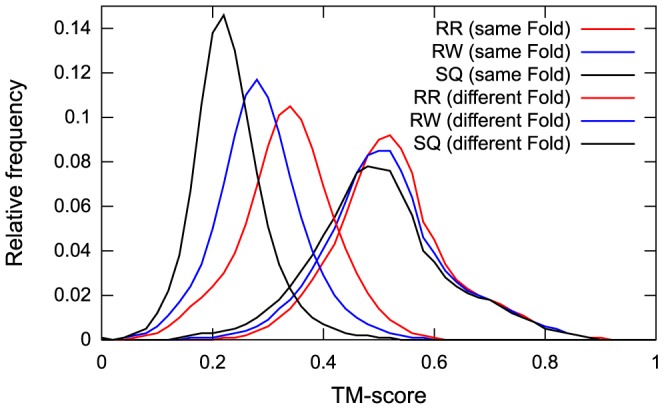
Comparison of the TM-scores among the best SQ, RW, or RR template and the corresponding query. The histograms of the TM-scores of the SQ, RW, and RR templates are represented as black, blue, and red lines, respectively.

Therefore, we performed structure alignment for a set of protein pairs sharing the same fold using the SQ, RW, and RR schemes of MICAN. As such a set, we chose a set of protein pairs of SCOP30 representatives with lengths between 80 and 200 amino acids in the same fold defined by the SCOP database, where SCOP30 consisted of SCOP domains with 30% maximum pairwise sequence identity. We refer to this dataset as the same fold set. Figure 12 shows the distribution of TM-score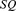, TM-score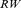, and TM-score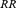 for the same fold set. In this calculation, the smaller protein in each pair was used as the target protein. The result illustrates that the distributions are quite similar to each other; the average values of (TM-score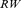 - TM-score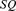), (TM-score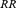 - TM-score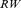), and (TM-score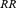 - TM-score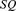) are 0.014, 0.010, and 0.024, respectively. The results for the same fold set sharply differed from those of a dataset consisting only of different fold pairs. To conduct comparative experiments, we performed structure alignments with the SQ, RW, and RR schemes of MICAN for a set of protein pairs belonging to different folds. This set, which we refer to as “the different fold set,” consists of protein pairs of the SCOP30 representatives classified in different folds. The distributions of TM-score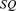, TM-score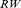, and TM-score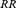 for the different fold set are shown in Figure 12. Clearly, the three distributions are significantly different; the average values of (TM-score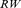 - TM-score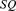), (TM-score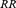 - TM-score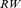), and (TM-score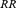 - TM-score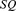) are 0.05, 0.06, and 0.10, respectively. These results suggest that the SQ, RW, and RR schemes of MICAN arrive at quite similar values of the TM-score for a protein pair with the same fold and sharply different values only for a protein pair with a different fold. In addition, the results suggest that, roughly speaking, a TM-score of 0.5 can be regarded as a reasonable threshold for sharing the same spatial arrangement of SSEs for the RW and RR schemes.

Around a TM-score of 0.5, we explored the TM-score threshold dependence of the network structure of the SQ, RW, and RR networks. Figure S2 shows the SQ, RW, and RR networks of various TM-score thresholds, ranging from 0.46 to 0.54. The figure shows that varying the threshold quantitatively changes the structures of the networks; as the threshold increases, the numbers of edges increase. However, it is also clear that varying the threshold does not change the structure of the network qualitatively in the range of 0.48–0.52. In this threshold range, The SQ network is mostly composed of isolated fold islands, except for some all-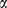 and 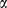/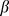 folds; the RW network has intra-class connections for all four classes; and the RR network has strong inter- and intra-class connections. Therefore, we conclude that a TM-score of 0.5 is an approximately reasonable threshold for sharing the same spatial arrangement of SSEs for all the SQ, RW, and RR schemes.

### A target protein set and structural similarity searches

We prepared target proteins, which were used as queries to search for similar SSE packing structures. The target protein set was derived from the fold representatives of the SCOP 1.75 database [33], which contains 1195 protein folds. The protein structures of the fold representatives were selected according to the ASTRAL compendium [53]. In our analysis, we eliminated small proteins (<40 residues or containing <3 SSEs) and proteins that primarily adopt coil conformations from the fold representatives. As a result, 1085 structures of SCOP fold representatives were included in the target protein set. For each target protein, we performed a structural alignments against the remaining 1084 target structures, which we called the template set of the target protein, and calculated the TM-score(target 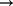 template) with the SQ, RW, and RR schemes using the MICAN program.

### The procedure of eliminating the contribution of protein folds that exhibit topological similarity to the target proteins

Here we describe the procedure of estimating the number of protein folds that share the same spatial arrangement of SSEs with at least one topologically different fold, eliminating the contribution of protein folds that exhibit topological similarity to the target proteins, using a TM-score threshold of 0.5. For a given target protein 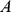, we searched for the template structure 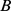 that satisfies the following two conditions: (i) the TM-score(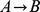) calculated by the RW or RR scheme is larger than 0.5 and (ii) the TM-score(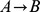) calculated by the SQ scheme is smaller than 0.5. If there was at least one template structure 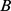 that satisfies aforementioned conditions, we regarded the target protein 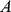 as having the same SSE packing arrangement with at least one topologically different fold. We repeated the same procedure for all of the target proteins and calculated the number of protein folds that share the same SSE packing with at least one topologically different structure.

## Supporting Information

Figure S1 **Target size dependence with various cutoff values.** The percentage of the target proteins with a TM-score of the SQ(A), RW(B), and RR(C) templates equal to or greater than various cutoff values as a function of the target protein size. The lines are colored red, yellow, black, green, and cyan for TM-score thresholds of 0.46, 0.48, 0.50, 0.52, and 0.54, respectively. The horizontal axis represents the sequence length of the target proteins. The vertical axis represents the percentage of the target proteins with TM-scores of SQ, RW, and RR templates equal to or greater than the TM-score threshold for a given target size.
(EPS)

Figure S2 **Graphical representation of the protein fold universe with various cutoff values.** The detailed graphical representations of the protein fold universe and the simplified networks drawn by the SQ, RW, and RR schemes with cutoff values of 0.46, 0.48, 0.50, 0.52, and 0.54.
(EPS)

Figure S3 **Relationship between the MCC value and the threshold.** The relationship between the MCC value to decide whether two structures are of the same fold, and the threshold for the SQ scheme of MICAN, TMalign, and HHsearch. The horizontal axis represents the threshold and the vertical axis represents the MCC value. As a dataset, we used the SCOP30 set.
(EPS)

Figure S4 **Graphical representation of the protein fold universe drawn by HHsearch, MICAN SQ, and TMalign.** The cutoff value of each method is determined by maximizing the MCC values to decide whether two structures are of the same fold using the SCOP30 set.
(EPS)

Text S1 **Comparison of the SQ network of MICAN with other methods.**
(PDF)
