# Supplementary material for: How a Spatial Arrangement of Secondary Structure Elements Is Dispersed in the Universe of Protein Folds
Source: PLoS One. 2014 Sep 22;9(9):e107959. doi: 10.1371/journal.pone.0107959 (PMC4171485; doi:10.1371/journal.pone.0107959)
Supplement: Text S1 — Comparison of the SQ network of MICAN with other methods. (PDF) [file pone.0107959.s005.pdf]

How a Spatial Arrangement of Secondary Structure Elements is Dispersed in the Universe of Protein Folds

Shintaro Minami[1], Kengo Sawada[2], George Chikenji[3,*]

1 Department of Complex Systems Science, Nagoya University, Nagoya, Aichi, Japan

2 Department of Applied Physics, Nagoya University, Nagoya, Aichi, Japan

3 Department of Computational Science and Engineering, Nagoya University, Nagoya, Aichi, Japan

Text S1

Comparison of the SQ network of MICAN with other methods

We showed in the main text that the SQ network is mostly composed of isolated fold islands, excluding some all-α and α/β folds. Some readers may have received the impression that the result of the SQ scheme is not consistent with those of previously published studies that support the concept of the continuity of the protein universe [1, 2]. Although the datasets as well as thresholds used in the previous studies were different from those used in ours, this inconsistency may give rise to a suspicion that the SQ scheme of MICAN is not sufficiently sensitive. This suspicion can be dispelled by comparison of the networks constructed by several methods using the same dataset and the threshold determined by the same criterion.

To make an appropriate comparison with previously published studies, we construct a graph representation of the protein fold universe by the SQ scheme of MICAN (MICAN SQ), TM-align [3], and HHsearch [4], using the same dataset and thresholds determined by the same criterion. The dataset we used here is the fold representatives of the SCOP 1.75 database, which is the same as that used in the main text. The thresholds of the three methods are determined by maximizing Matthews correlation coefficient (MCC) to decide whether two structures are of the same fold using the SCOP30 dataset. Figure S3 shows the relationship between the MCC value and the threshold for the three methods. The thresholds that maximize the MCC values are a TM-score of 0.48, TM-score of 0.52, and p-value of 10^{-4} for MICAN SQ, TM-align, and HHsearch, respectively. We used these threshold values to construct the protein universe graph.

Figure S4 shows the graph representations of the protein fold universe and their simplified networks connected by HHsearch, the SQ scheme of MICAN, and TM-align, respectively. As described in the main text, in the simplified networks, if $\overline{N_X^{\mathrm{edge}}(A \to B)}$ is larger than 1.0, we drew directed edges between the nodes and presented its numerical value near the edge. The network of MICAN SQ shown in Figure S4 is qualitatively the same as the SQ network shown in Figure 10, although the cutoff values are slightly different; a TM-score of 0.48 is used in Figure S4 and a TM-score of 0.50 in Figure 10. It is obvious that the network of HHsearch is much more disconnected than that of MICAN SQ, implying that MICAN SQ is much more sensitive than HHsearch. The network of TM-align, in contrast, is visually quite similar to that of MICAN SQ; both networks are mostly composed of isolated fold islands, excluding some all-α and α/β folds. These results suggest that the MICAN SQ scheme is reasonably sensitive.

References

1. Skolnick J, Arakaki A, Lee S, Brylinski M (2009) The continuity of protein structure space is an intrinsic property of proteins. Proc Natl Acad Sci USA 106: 15690–15695.

2. Alva V, Remmert M, Biegert A, Lupas A (2010) A galaxy of folds. Protein Sci 19: 124–130.

3. Zhang Y, Skolnick J (2005) TM-align: a protein structure alignment algorithm based on the TM-score. Nucleic Acids Res 33: 2302–2309.

4. Söding J (2005) Protein homology detection by HMM-HMM comparison. Bioinformatics 21: 951–960.
